# Age-Dependent Geographic Convergence of Overdose and Cardiovascular Emergency Medical Services Calls: A Spatial Point Process Analysis

**DOI:** 10.5888/pcd23.250450

**Published:** 2026-07-30

**Authors:** Jeffrey Pesarsick, Ruchi Bhandari, Evonne Richards, Caroline Groth, Scott Findley, Brian Hendricks

**Affiliations:** 1 West Virginia School of Osteopathic Medicine, Center for Rural and Community Health, Lewisburg, West Virginia; 2 West Virginia University, Epidemiology and Biostatistics, Morgantown, West Virginia; 3 West Virginia University School of Medicine, Emergency Medicine, Morgantown, West Virginia

## Abstract

**Introduction:**

Overdose and cardiovascular disease are both leading causes of emergency medical services (EMS) activation. The objective of this study was to identify differential patterns in cardiac EMS activations by age and explore whether suspected overdose activity influenced these differences.

**Methods:**

EMS activations data were obtained using emsCharts software for Washington County, Pennsylvania, from 2017 through 2023. Poisson point process model (PPM) regression analyses were conducted to estimate the cardiac EMS activation per unit area, as a function of age and suspected overdose, adjusting for population size.

**Results:**

PPM regression analyses identified significant differences in EMS cardiac activations for people aged 18 to 45 years compared with people older than 45 years (β = −1.83, SE = 0.020, 95% CI = −1.86 to −1.79). EMS activations increased as population size increased (β = 0.21, SE = 0.001, 95% CI = 0.21 to 0.22). Analysis also identified significant differences in EMS activation per unit area for age groupings, depending on local suspected overdose activity (interaction term) (β = 0.02, SE = 0.001, 95% CI = 0.01 to 0.02). Likelihood-ratio tests indicated that using suspected overdose activity as an effect modifier on age-dependent cardiac EMS activations improved model fit (deviance = 228.12, *df* = 1, *P* < .001).

**Conclusions:**

The community-level occurrence of cardiac emergencies differs by age group, with younger adults experiencing more cardiovascular disease in neighborhoods with higher suspected overdose activity. This nuance is key to developing age-appropriate public health intervention materials to lessen early onset cardiovascular disease illness and death.

**Summary**

**What is known on this topic?**

Cardiac-related emergency medical services (EMS) activations differ depending on the rate of substance use and population demographics.

**What is added by this report?**

Previous studies are missing the spatial analyses needed to quantify the extent to which substance use influences differential clustering of cardiac EMS activations by age.

**What are the implications for public health practice?**

Findings are key to implementing age-appropriate public health education to prevent early onset heart disease and demonstrating how spatial analyses can be applied to help inform health decision making.

## Introduction

Cardiovascular disease (CVD) is the leading cause of death in the US, with a crude mortality rate of 202.5 deaths per 100,000 population (1–3). Heart attack and stroke incidence among emergency medical services (EMS) responses represents approximately 12% of EMS activations and costs the US health care system approximately $240 billion annually (3–5). Risk factors for CVD include health conditions, such as diabetes, as well as lifestyle behaviors including smoking, low physical activity, poor diet, and substance use disorder (6–9).

Illicit drug use is associated with increases in early onset CVD, overdose, and death (9–15). One in 5 young adults engages in polysubstance use, with the odds of CVD increasing incrementally with the number of substances used (13–15). Although current research demonstrates the rate of CVD in adults aged 50 years or older is declining, the rates of obesity, diabetes, and substance use disorder in adults younger than 50 years are increasing (16,17). A recent Centers for Disease Control and Prevention (CDC) report concluded that overdose risk was highest for US adults aged 35 to 44 years, with decreased risk observed for people aged 45 years or older (18).

The overlap in age groupings for historically low risk of CVD and high risk of drug overdose could help explain rising trends in cardiac EMS activations among younger adults. Previous studies using national EMS data found that substance use–related cardiac EMS activations increased in communities on the basis of their population’s demographic makeup (19,20). However, no study to date has examined how suspected overdose activity influences where EMS activations for cardiac events occur for different age groups. The goal of this study was to identify differences in where cardiac EMS activations occur by age group and whether suspected overdose activity has a differential effect on the frequency of the activations within age groups.

## Methods

### Data management

Address-level, geocoded EMS activations data for 2017 through 2023 were obtained for Washington County, Pennsylvania, from emsCharts (ZOLL Data Systems). Data were requested from Washington County Ambulance and Chair EMS, who serve the 34 municipalities that cover 487 square miles and 101,000 residents living in Washington County, Pennsylvania. This study was approved by the West Virginia University Institutional Review Board (protocol no. 2512261211).

The outcome for the analysis was defined as intensity (EMS activations per unit area) with indicators of a CVD complaint in the impression. The impression field is where EMS providers document their primary assessment of the patient’s most significant condition or conditions. Indicators of cardiac EMS activations included “stroke,” “chest pain,” “hypertension,” “cardiac arrest,” “myocardial infarction,” “cardiac rhythm disturbance,” and “aortic dissection” complaints. Predictors included 1) age of each cardiac EMS activation coded as a binary variable for age grouping (18–45 y or >45 y). Age 45 years was used as a threshold in which we anticipated more frequent drug involvement, as concluded in a recent CDC report that indicated that people aged 34 to 45 years had the highest risk of overdose (18). Other predictors included suspected drug overdose among all EMS activations where the primary or secondary impressions were “poisoning/drug ingestion” and the chief complaint was overdose or contained related key words, such as “heroin overdose,” “possible prescription drug overdose,” “not breathing, possible overdose,” “overdose,” “overdose, unresponsive,” “dead on arrival, drug paraphernalia present,” “unresponsive, IV drug use per patient.”

To limit bias in EMS activations, we adjusted for population size at the census block level. Census block–level population data were obtained from the Esri Living Atlas and visualized in ArcGIS Pro (Esri); the Polygon to Raster tool was used, setting cell size to population size (21). Next, the cardiac EMS activations were visualized as points, and the Extract Values to Points tool was used to extract the predicted population size at the activation latitude and longitude (22). Population size and the binary age variable were contained in the same data set as the latitude and longitude for cardiac EMS activations. Suspected overdose activity among all EMS activations for the study period and area were available in a separate data set containing only latitude and longitude.

### Analysis

Marked point pattern “ppp” objects were created for cardiac EMS activations, population size at the cardiac EMS activation site, and suspected overdose among all EMS activations using latitude, longitude, and Washington County boundary as the spatial window (eg, boundary). Before the shapefile for the county boundary could be used, it had to be converted into a 2D planar object using the st_transform function with UTM zone 17 specified. The cardiac EMS activations “ppp” object included the binary age grouping as a mark. A mark in spatial point pattern analysis is a label or characteristic attached to each point, like type of event or age group. Having age as a mark in the “ppp” object for cardiac EMS activations allowed us to conduct an age-stratified analysis of their intensities.

Point density or spatial image objects were estimated for cardiac EMS activations and suspected overdoses through the density.ppp function, where the smoothing bandwidth was chosen using Diggle’s cross-validation method to minimize mean square error (prediction error) in observed point pattern (23). The spatial image object for population size was computed using kernel regression to estimate the expected population value at each location using the nearest neighbors algorithm (KNN = 3). The spatial image object for population size was created using a different approach from suspected overdose or EMS cardiac activations because the image was based on the value of the population size at each centroid, as opposed to just where the event occurred.

Separate point process models (PPMs) were conducted using the ppm function to 1) identify age-dependent cardiac EMS activations per unit area and 2) evaluate the effect of an age-by–suspected overdose interaction term on cardiac EMS activation intensity. Both models included population size to adjust for the probability of more EMS activations in more populated areas. Both predictors (population and suspected overdoses) were standardized by calculating *z*-scores to create unitless covariates with comparable effect sizes. As a measure of sensitivity, another PPM was conducted including only main effects for age grouping, population size, and suspected overdose activity (eg, no interaction term for age by overdose activity). Likelihood ratio tests were conducted to examine the impact of stratifying by age and finally adding suspected overdose–by-age interaction terms on model performance. Model performance was assessed through deviance at 1 degree of freedom. All statistical significance was assessed at the 2-sided 0.05 α level and 95% CI. All statistical analyses were conducted in RStudio and used the sf, spatstat.explore, spatstat.model, spatstat.geom, and lwgeom packages (24–27).

## Results

A total of 89,698 EMS activations occurred during the study period. Of these, 28,620 (32%) had impressions consistent with our definition of a cardiac EMS activation. Among cardiac EMS activations, 293 (1.0%) were removed due to being outside the study area. Of the 28,327 remaining cardiac EMS activations, 24,400 (86.1%) were for people older than 45 years, and 3,930 (13.9%) were for people aged 18 to 45 years. The mean intensity for cardiac EMS activations among people older than 45 years was 1.09 × 10^-5^ events per meter squared compared with 1.75 × 10^-6^ events per meter squared for people aged 18 to 45 years. These findings translate to 28 cardiac EMS activations every square mile for people older than 45 years compared with 5 cardiac EMS activations among those aged 18 to 24 years.

**Figure 1:**
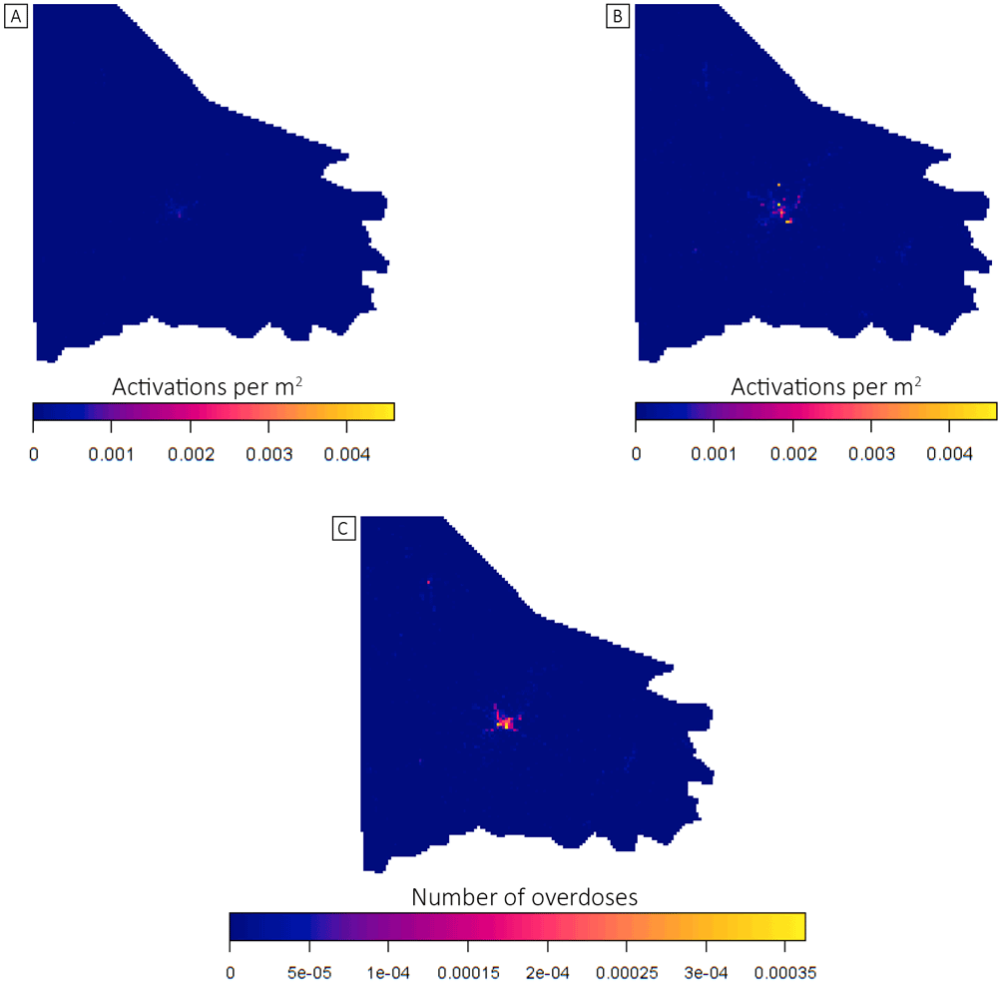
Cardiac EMS activations per meter squared, by patients aged 18 to 45 years (graph A) and older than 45 years (graph B), and suspected overdoses among all EMS activations (graph C), Washington County, Pennsylvania, 2017–2023. EMS, emergency medical services.

**Figure 2:**
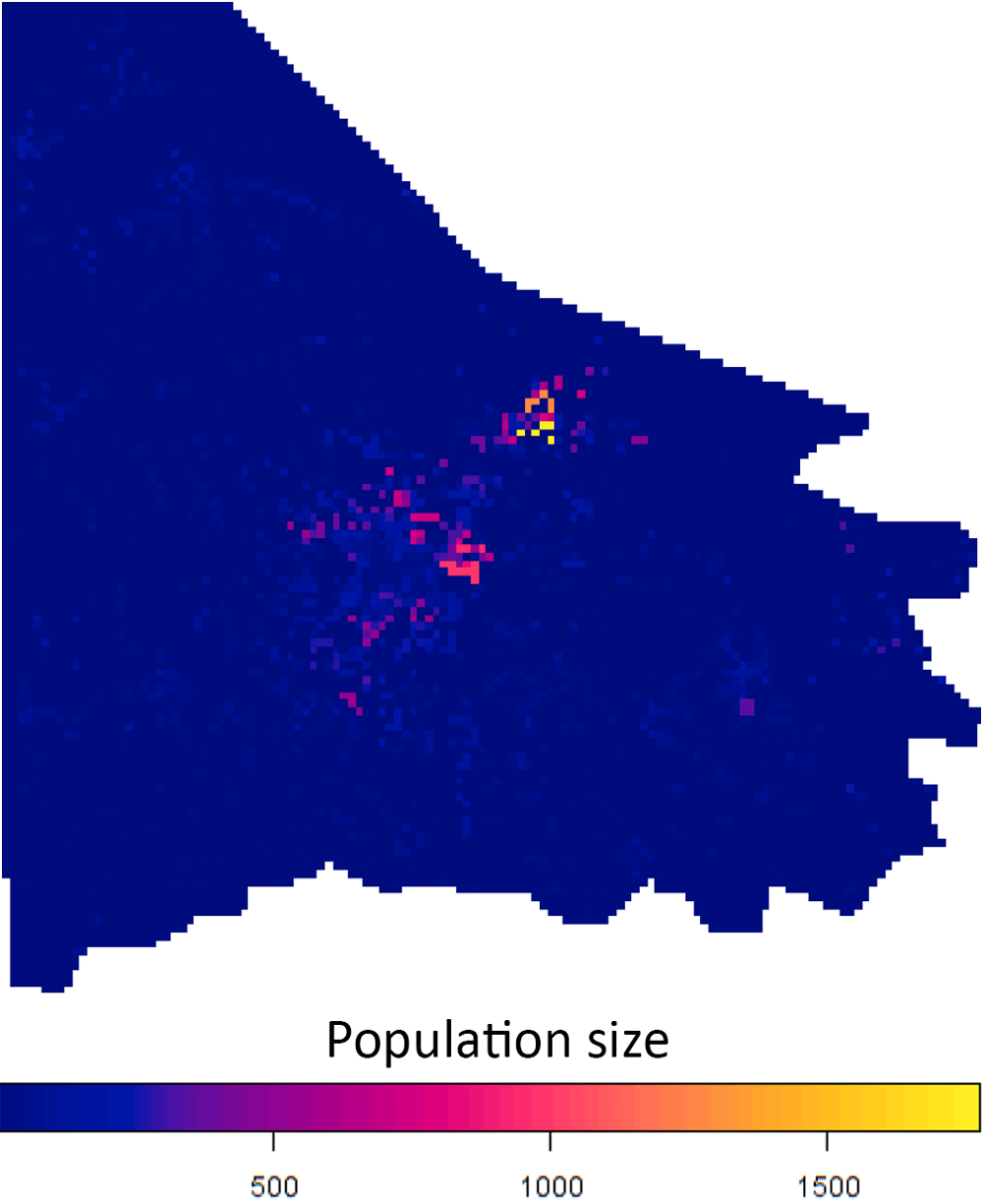
Raw kernel regression smoothed maps for population per meter squared in Washington County, Pennsylvania, using census block population estimates, 2025.

EMS activations for suspected overdoses during 2017–2023 totaled 2,061. Of these, 17 were outside the study area and excluded from analysis. The mean intensity of suspected overdoses among all EMS activations was 9.09 × 10^-7^ events per meter squared. These findings translate to 2 suspected overdoses among all EMS activations per square mile for Washington County, Pennsylvania. Raw point density maps for cardiac and suspected overdose EMS activations are displayed in Figure 1. Raw kernel regression maps for population size are displayed in Figure 2.

Cardiac EMS activations were higher for people older than 45 years compared with those aged 18 to 45 years and increased with population size (Table 1). Findings indicated that cardiac EMS activations increased by 23% for every 1 standard deviation increase in population ([1 − exp(0.21)] × 100). Population-adjusted age-related cardiac EMS activations were 83% less likely for people aged 18 to 45 years, compared with those older than 45 years ([1 − exp(−1.83)] × 100). 

**Table 1:** Point Process Model Regression Analysis, Age Group Effect on Cardiac Emergency Medical Services Activations in Washington County, Pennsylvania, 2017–2023

Variable	Model estimate (95% CI)	Standard error
Aged 18–45 y	−1.83 (−1.86 to −1.79)	0.020
Population size	0.21 (0.21 to 0.22)	0.001

Cardiac EMS activations differed by age group and were influenced by suspected overdose activity, after adjusting for population size (Table 2). Every 1 standard deviation increase in suspected overdose EMS activations was associated with an increased intensity of cardiac EMS activation among people aged 18 to 45 years by 2% compared with those older than 45 years ([1 − exp(0.02)] × 100). 

**Table 2:** Point Process Model Regression Analysis, Age Group by Suspected Overdose Interaction Effect on Cardiac Emergency Medical Services Activations in Washington County, Pennsylvania, 2017–2023

Variable	Model estimate (95% CI)	Standard error
Aged 18–45 y	−1.96 (−2.00 to −1.92)	0.020
Population size	0.21 (0.21 to 0.22)	0.001
Suspected overdoses	0.18 (0.17 to 0.18)	0.001
Age*overdose interaction	0.02 (0.01 to 0.02)	0.001

The PPM with the interaction term performed statistically better than the PPM with main effects only (ie, no interaction term for age by overdose activity) (deviance = 228.12, *df* = 1, *P* < .001) (Table 3).

**Table 3:** Point Process Model Regression Analysis, Age Group and Suspected Overdose Effects on Cardiac Emergency Medical Services Activations in Washington County, Pennsylvania, 2017–2023

Variable	Model estimate (95% CI)	Standard error
Aged 18–45 y	−1.827 (−1.860 to −1.793)	0.020
Population size	0.215 (0.212 to 0.217)	0.001
Suspected overdoses	0.183 (0.182 to 0.184)	0.001

## Discussion

According to our knowledge, this study is the first to apply point process modeling methods to ascertain the geographic context of age-related CVD disparities. In this spatial epidemiologic study using EMS activation data, we found that occurrence of CVD in the community differed based on population size and patient age. More specifically, cardiac EMS activations occurred 84% less among people aged 18 to 45 years compared with those older than 45 years. However, an interesting finding was that the areas with increased co-occurrence of suspected drug overdoses had the higher occurrence of CVD-related EMS activations for people aged 18 to 45 years. Importantly, we adjusted for population size to mitigate the effect of communities having more calls due to their larger population size.

Our results corroborate past nonspatial epidemiologic investigations using national EMS data sets (19,20). These studies also found that increased CVD disease risk is associated with history of past drug use. Our study adds to this body of research by exploring neighborhood-level interactions between suspected drug activity and heart disease risk across the age spectrum, which may be particularly relevant for states like West Virginia, Kentucky, and parts of Pennsylvania included in the Appalachian region that are at increased risk of drug-related deaths related to the sole use of mixed-use of opioids with other substances (eg, methamphetamine, alcohol) (20,28,29). These states all experience similar trends in premature CVD mortality rates with increasing adjusted rates in rural and socially vulnerable counties (30,31). Our study area of Washington County, Pennsylvania, has urban and rural communities mixed in its municipalities. It is possible that the 2% increase in cardiac EMS activations among people aged 18 to 45 years for each standard deviation increase in suspected overdoses could differ within communities that fall within the rural–urban divide due to resourcing, access to care, and public health intervention gaps identified by past research.

The interaction of age and suspected overdose activity among early CVD has public health implications. The spatial overlap may reflect clustering of acute health vulnerabilities in certain communities, suggesting plausible increased early CVD in younger populations. This finding supports the integration of CVD prevention messaging in harm-reduction programs. Importantly, this research supports the use of EMS activation data as a public health tool, where EMS activation data could be used in real-time surveillance models for identifying neighborhoods with overlapping heath burdens.

There are limitations to our approach. First, the EMS activation data were not linked to hospital medical records, preventing confirmatory diagnoses of CVD or overdoses. Instead, we leveraged the responding EMS provider impressions to parse data down to our analytic sample. Early CVD is not solely propagated through substance use disorder or substance misuse; it is a complex condition with a multitude of risk factors such as tobacco use, physical inactivity, and more (16). These other risk factors were not included in our study or the EMS activation data set. This study was conducted in Washington County, Pennsylvania, and the spatial patterns observed may not be representative of a broader geographic region which may differ in population, demographic profile, and health care access. Despite these limitations, our approach has several strengths, one of which is the use of address-level EMS activation data. The use of sophisticated point process regression modeling, which provided a granular community-level interpretation of cardiac EMS activations among age groups, is also a strength. All analyses were population adjusted, and predictors were standardized to yield comparable effect sizes. These methods all contribute to clarity in interpreting the study findings and its reproducibility elsewhere.

Previous research demonstrates an increasing rate of early onset CVD and declining risk among older populations (14,16), which could suggest that our policy and public health responses also need to shift. Beyond typical CVD education, efforts should focus on the risk factors, such as substance abuse and its co-occurring mental illnesses, affecting people below the normal threshold of age thought to be at risk for heart disease. Spatial analysis is a powerful tool that can help direct education and prevention efforts at the community level and guide state and federal policy. EMS agency data often lack formal diagnoses, but their benefits to improving community health and wellness should not be overlooked. Few public health data sources are available at the same spatial resolution. More research is warranted to validate the spatial patterns identified through follow-up observational research that explores community-based causal pathways and to measure the effect of population-based interventions on reducing the risk of early stage CVD through closing rural–urban gaps in co-occurring conditions, like substance use disorder and substance misuse. More specifically, future point process analysis could be applied to larger regions or include more patient-specific data, such as CVD risk factors or other relevant overdose risk data. Incorporating more patient-specific data at the site of cardiac EMS activations would strengthen this approach and its ability to guide community response or public health intervention.
